# Citizen science in plant disease surveillance

**DOI:** 10.3389/fpls.2026.1775094

**Published:** 2026-02-05

**Authors:** Amelia Martin, Nicholas Goltz

**Affiliations:** UConn Plant Diagnostic Laboratory, Department of Plant Science and Landscape Architecture, University of Connecticut, Storrs, CT, United States

**Keywords:** community initiative, disease detection, disease monitoring, plant health, qualitative data

## Abstract

Plant diseases cause an estimated 30% annual crop yield loss worldwide, resulting in hundreds of billions of dollars in economic damage and increasingly threatening native and ornamental plants through global trade and environmental change. In the United States, the National Plant Diagnostic Network (NPDN) plays a critical role in safeguarding agricultural biosecurity; however, limited personnel and uneven diagnostic capacity highlight the need for new approaches to enhance disease surveillance. Citizen science, defined as public participation in scientific data collection, has proven effective in ecology and environmental monitoring but remains underutilized in plant pathology, prompting the need to assess its applicability in this field. This study represents the first national effort to evaluate citizen scientists’ perspectives on participation in plant disease research. To address this objective, a nine-question online survey was distributed to approximately 500 individuals, including Master Gardeners, Extension professionals, and plant enthusiasts, yielding 233 completed responses. Results indicated strong interest in contributing to plant disease data collection, with participants emphasizing the importance of accessible education, diagnostic guidance, and user-friendly reporting platforms. Although challenges related to diagnostic accuracy and potential user bias persist, the findings suggest that structured training, educational support, and professional validation could enable citizen science to become a valuable tool for expanding plant disease surveillance. To the extent of our knowledge, this work provides the first evidence-based insight into citizen scientists’ readiness to engage in plant disease monitoring and offers practical guidance for developing inclusive and effective plant health programs.

## 1 Introduction

Plant diseases are among the most significant threats to global agriculture, causing an average annual crop yield loss of approximately 30% and resulting in economic losses estimated in the hundreds of billions of US dollars (Gai and Wang, 2024). Outside of agricultural production, emerging infectious plant diseases are a major problem to native trees and non-native plants bred for aesthetics and forestry (Gougherty, 2023). Recent studies indicate that both endemic and emerging plant diseases are spreading at an unprecedented rate, driven by factors such as varying weather conditions, global trade, and increased movement of plant materials (Ristaino et al., 2021). At the forefront of plant disease detection and management is the National Plant Diagnostic Network (NPDN), founded by the United States Department of Agriculture (USDA) after the events of September 11^th^, 2001. The primary goal of the NPDN has been to protect the biosecurity of agriculture within the United States by providing accurate and timely plant diagnostics (NPDN, 2025, “History of NPDN”). Today, the work of the NPDN is conducted through plant diagnostic laboratories within each of the 50 US states, the majority based at land-grant universities. While the threat of emerging plant diseases escalates, so do challenges for the plant diagnosticians. With limited trained personnel working within each plant diagnostic laboratory, and resources varying between different sites, it is critical to develop new technologies and methods to expand the early detection and surveillance of plant pathogens.

Throughout history, one practice that has yielded successful results across various fields in biology, ecology, and healthcare is the use of citizen science. Citizen science is the practice of public participation and collaboration in scientific research to increase scientific knowledge (Ullrich, 2024). Across all fields, the central goal of citizen science remains consistent: to expand the scope and volume of data collection through community involvement. While the practice spans many disciplines, it is especially common in projects related to the natural world, with one of the earliest well documented examples being the Christmas Bird Count conducted by the Audubon Society in 1900 (Zukauskas, 2024). Since then, citizen science has gained a newfound popularity, with some examples being contributing to astrological data collected by NASA (NASA, 2025) and the monitoring of marine debris movements by National Oceanic and Atmospheric Administration (NOAA Marine Debris Program). As citizen science initiatives continue to increase, one discipline that remains slow to integrate is plant pathology.

Citizen science is not a novel approach in plant science with long-standing initiatives already generating valuable data on plant phenology and plant-animal interactions (Chicago Botanic Garden, Adkins Arboretum). While there are many identification projects utilizing citizen science in plant science, few projects utilizing the approach in plant disease research exist, with the most successful being the discovery of hidden phytoplasmas (Brochu et al., 2023). The sentiment of incorporating citizen science in plant disease research is well thought out and desired, with previous studies highlighting the innovative role citizen science could contribute to the field (Brown and Williams, 2019). However, as promising as citizen science may be for increased plant disease surveillance, collecting and reporting accurate data on plant diseases presents unique barriers. Among these barriers, the most challenging is the accurate identification of the causal agent, as diagnostic interpretation may vary due to diseases producing a wide range of symptoms, different diseases exhibiting similar symptomatology, variation in individual plant history, and differences in the experience level of the identifier (Barbedo, 2016). These factors combined push accurate plant diagnostics past solely visual assessments as it often requires additional laboratory testing conducted by trained personnel. While these challenges pose significant barriers to adopting citizen science in plant disease research, citizen science still remains a viable tool to try in the future, so long as the correct foundational work is conducted.

With no prior research conducted directly engaging citizen scientists in the field of plant disease, this study seeks to fill that critical gap by providing insights that may set forth a framework or increase of understanding for plant pathologists looking to utilize citizen scientists. To do this, a national nine question online survey was distributed to extension professionals, plant groups, and clubs across the U.S. with the goal of eliciting responses from anyone interested in plants from a diverse set of educational backgrounds. The main project objective was to evaluate the potential of citizen science as a tool for expanding early detection and reporting of plant diseases. To meet this objective three broad goals were established: (1) to assess participant’s plant disease knowledge, willingness to participate, and citizen science background, (2) to identify what obstacles exist to integrating citizen science in the field of plant pathology, and (3) to identify best methods of utilizing and supporting citizen science in plant disease surveillance. The survey collected no identifiable information and collected a mix of quantitative data through multiple choice selections, and qualitative data through open-ended answers. Survey responses were analyzed through thematic organization and systematic coding to identify viable pathways for engaging citizen scientists in plant disease research, as informed directly by the target audience.

## 2 Materials and methods

### 2.1 Experimental design

The study design included two primary phases: a pilot trial survey and an official online survey. The aim was to evaluate the potential of citizen scientists as a tool for expanding the early detection and reporting of plant diseases and identify ways to leverage this.

This study used a mixed methods design which involved multiple choice and open-ended questions as a way to gain both quantitative and qualitative data (Creswell and Hirose, 2019). The study design reflected the project goal of collecting data from a wide audience while ensuring a nuanced understanding of their responses, while also following the standard for design for online surveys (Wasti et al., 2022). The first project goal, to assess participant’s plant disease knowledge, willingness to participate, and citizen science background was divided into three question topics from which all survey questions were created under. Nine survey questions were created and organized under appropriate topic areas. Of the nine survey questions, only two were open-ended, with the remaining questions being multiple-choice, in order to minimize missing data and maintain response quality typically reduced by open-ended items (Reja et al., 2003). The study phases were structured sequentially to validate and refine data collection methods before releasing the survey to a wider audience. The pilot trial survey contained identical questions to the official online survey and was conducted voluntarily with a control group of 15 self-described citizen scientists, at an unrelated in person event at the University of Connecticut. Once the initial survey was validated through positive user feedback, pilot responses were omitted as to not skew nationwide survey data, and the official survey was published. The survey remained open for 21 days and survey was disseminated through all chosen communication channels on the same day.

The online survey was conducted via Qualtrics, 2025 (https://www.qualtrics.com), and participants were recruited via email invitation or shared QR code link, through national extension listservs, such as the National Plant Diagnostic Network listserv, and the American Phytopathological Society (APS) listserv. A flyer was also created and disseminated via LinkedIn. The survey included nine questions consisting of six in multiple-choice and three in open-ended format (**Table 1**). The estimated time to complete the survey was approximately 5–10 minutes, which follows the recommended time length before survey fatigue and dropout (Revilla and Ochoa, 2017).

**Table 1:** Survey topics and corresponding questions.

Survey topics	Questions
Demographic Data	Q.1 What category do you most identify with? [multiple choice]
1. Plant Disease Knowledge	Q.2 Rate your knowledge on plant diseases [multiple choice]
	Q.3 Where did you learn about plant diseases? [multiple choice]
	Q.4 Would you be interested in learning more about plant diseases? [multiple choice]
2. Citizen Science Background	Q.5 Have you ever shared nature photos to online groups? [multiple choice]
	Q.6 If yes, to what platforms? [open ended]
	Q.7 If no, why not? [open ended]
3. Willingness to Participate	Q.8 If there was a way to share plant disease findings to researchers, would you be open to it? [multiple choice]
	Q.9 Any ideas, suggestions, or comments regarding citizen science in plant disease surveillance? [open ended]

### 2.2 Ethical considerations

Before conducting any research activities, approval was received from the University of Connecticut Institutional Review Board (IRB) and from the Biomedical Research Alliance of New York (BRANY) to ensure that the inquiry of human subjects was conducted in accordance with legal requirements and ethical principles of Respect for Persons, Beneficence and Justice.

Participation was voluntary; informed consent was provided on the first page of the survey. Completion of the survey indicated consent. Participants could withdraw from the study at any time without penalty by discontinuing (not submitting) the online survey or, after completing the survey, by contacting the study researchers to request that their response not be included. No identifiable information was collected, and all data is only accessible to research staff.

### 2.3 Participants

This study does not include a formal screening process; instead, participation is based on self-selection and self-reporting following the recruitment message and informed consent. Individuals who review the consent statement and elect to complete the online survey are considered enrolled upon initiation. Participation is voluntary and open to adult citizen scientists in the United States, including Master Gardener volunteers, STEM educators, and extension professionals. To minimize bias, recruitment material was standardized, and recruitment materials were distributed across multiple channels to ensure a broad representation. The survey did not ask for identifiable information such as name, race, ethnicity, or gender.

The survey was distributed to approximately 400 potential participants through email and posted to online groups and via LinkedIn for an additional goal of 100 viewers, with an anticipated 100–150 completed responses (30% response rate). This sample size was appropriate for the exploratory and formative nature of the research and sufficient to detect descriptive patterns. Attrition risk is minimal, though up to 15% of responses were expected to be incomplete (Hoerger, 2010).

Eligibility criteria included adults aged 18 years or older who are located within the United States, have an affiliation or interest in plant science or agriculture, and possess the means to engage with citizen science activities (e.g., access to technology or a camera). Eligibility was confirmed through self-screening. Following guidance from University of Connecticut Internal Review Board (IRB), exclusions included individuals under 18, prisoners, and those residing outside the United States.

**Figure 1:**
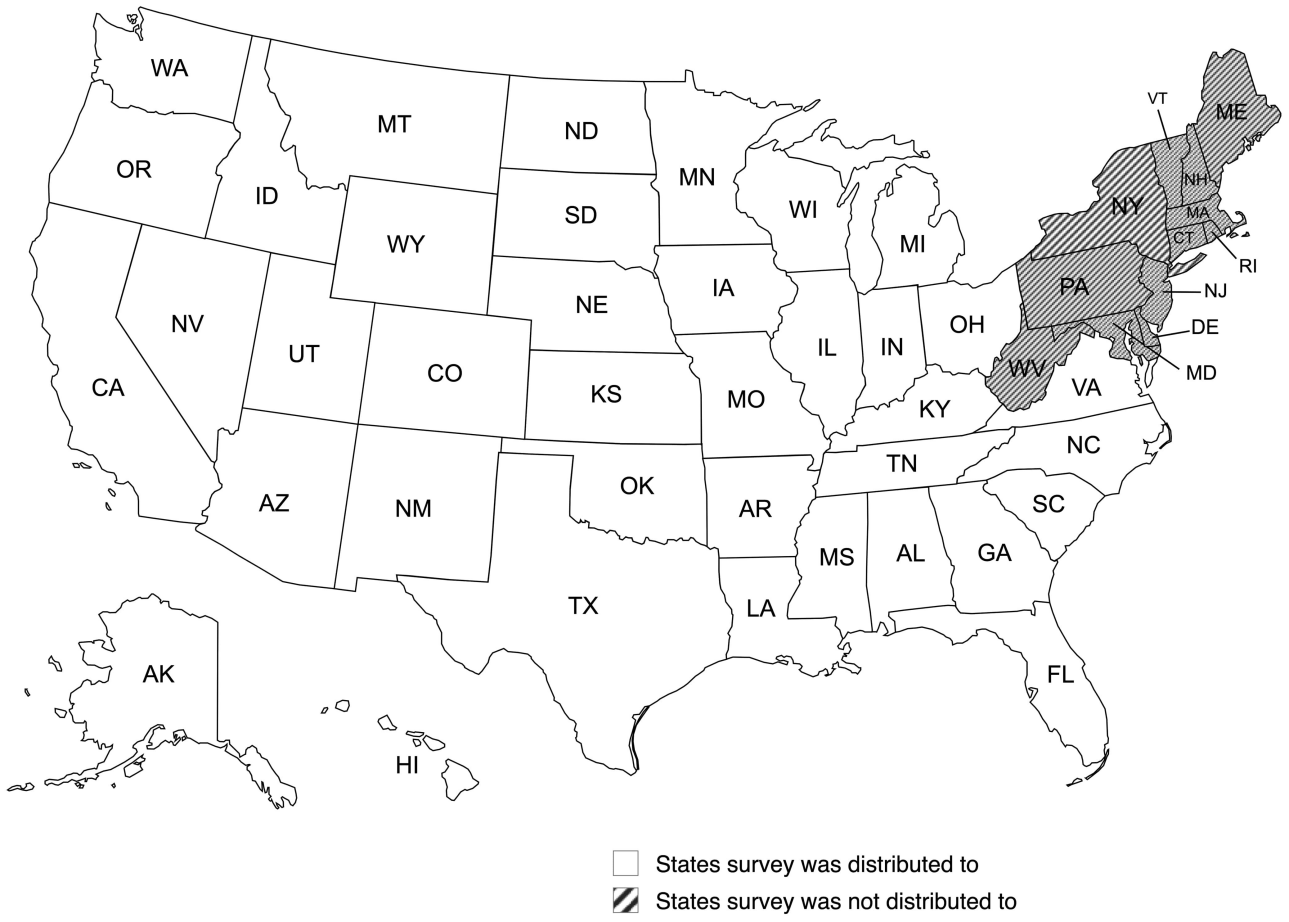
Map of U.S. states survey was and was not distributed to.

While participants located anywhere within the United States were eligible to participate in the survey, it was distributed only to states that were not located in the Northeast region of the United States (**Figure 1**). This is because a second study focusing solely on the Northeast region was conducted at the same time. With both studies including an online survey on different subjects matters needing to be distributed by extension professionals to the same stakeholders- the region was excluded as to not overload any voluntary survey participants.

### 2.4 Data collection and analysis

This study used one primary tool for data collection: a researcher-developed online survey via Qualtrics. This instrument is designed to align with the study’s purpose of exploring the potential of citizen scientists as a tool for expanding early detection and reporting of plant diseases. A report for the online survey data was generated following the survey’s closure using Qualtrics’ built-in reporting tools. The data were organized into tables for multiple-choice responses and compiled into categorized lists for open-ended questions. Multiple-choice tables were reviewed for patterns while open ended data were reviewed and analyzed for reoccurring words or trends.

## 3 Results

### 3.1 Survey participants and topics

Of the estimated 500 individuals invited to participate, 267 initiated the survey and 237 proceeded beyond the informed consent page. The highest number of completed responses for any single item was 233, which was therefore used as the final sample size for analysis. Qualitative data was collected through open-ended questions, coded, and categorized. Open-ended data is displayed by category and number of respondents, while quantitative data was collected for all others and displayed as figures.

**Figure 2:**
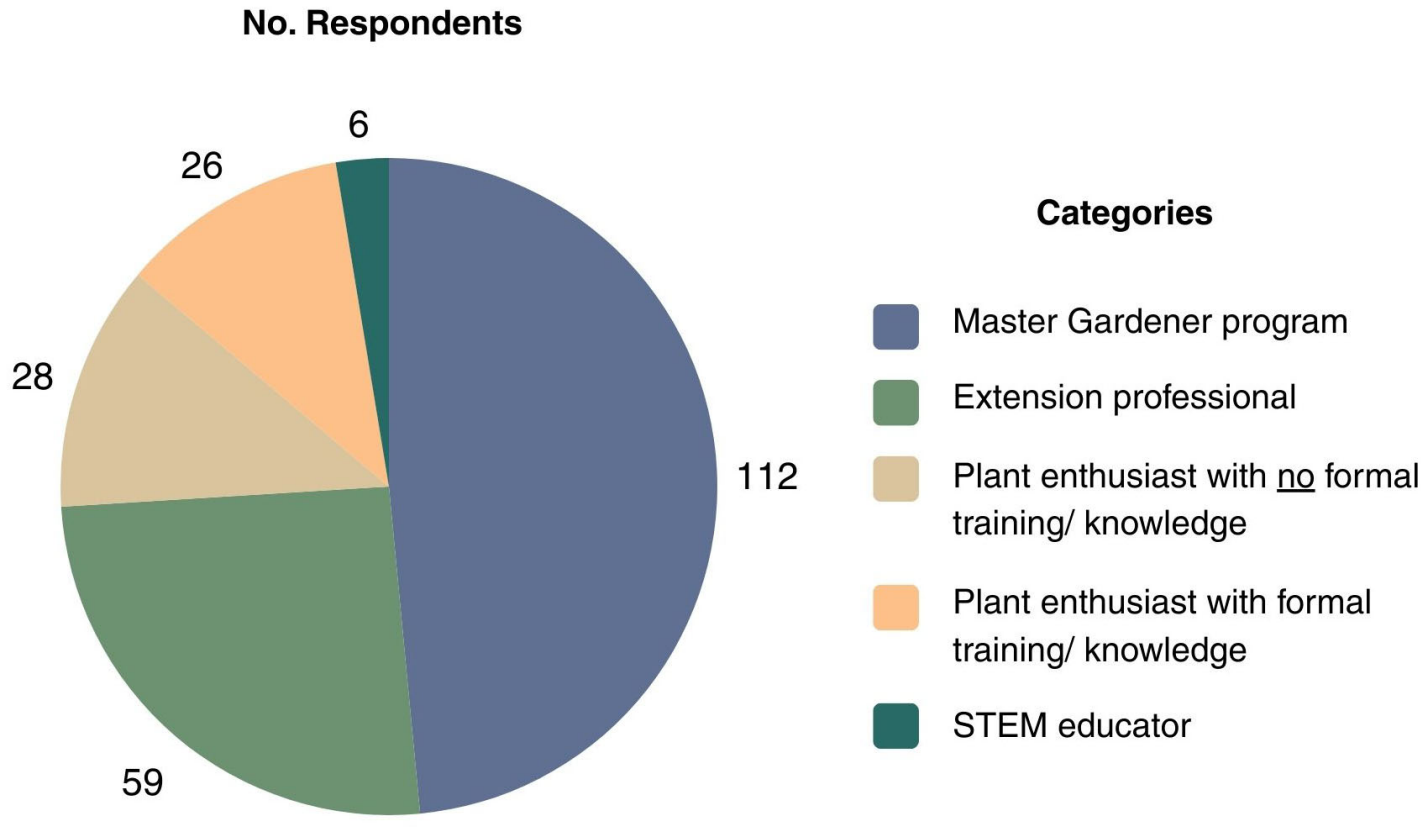
Question 1 (Q1) data on survey respondent roles.

The first survey item (Q1) collected participant demographic information, specifically the role with which respondents most identified within the plant sector. Of the 231 responses, 112 (48.5%) identified with the ‘Master Gardener program’, followed by 59 (25.5%) who identified as ‘Extension professionals ‘(**Figure 2**).

### 3.2 Topic results

#### 3.1.1 Plant disease knowledge

**Figure 3:**
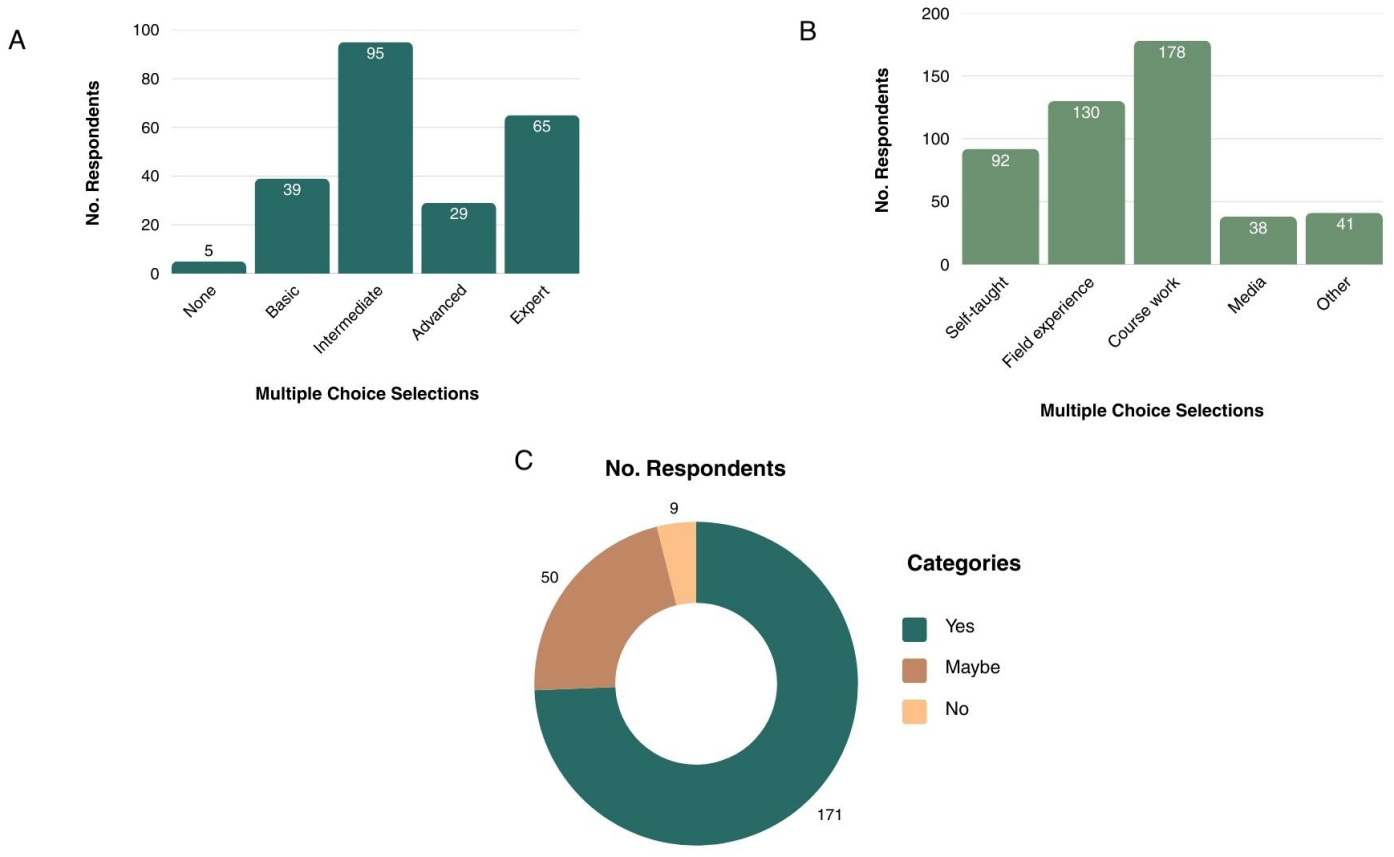
**(A)** Question 2 (Q2) data on survey respondents’ current plant disease knowledge level. **(B)** Question 3 (Q3) data on where survey respondents learned about plant diseases. **(C)** Question 4 (Q4) data on if survey participants would like to have learn more about plant diseases given the opportunity.

Participants were asked to rate their understanding of plant diseases (Q2). Among the 233 respondents, 95 (40.8%) indicated intermediate knowledge, and 65 (27.9%) described their knowledge as “expert” (**Figure 3**). Participants were next asked to identify all applicable sources from which they had learned about plant diseases (Q3). The most frequently selected source was coursework (n = 178), followed by field experience (n = 130) and self-teaching (n = 92). A total of 230 respondents answered when asked whether they were interested in learning more about plant diseases (Q4). The majority of participants responded yes (n = 171, 75.7%), followed by maybe (n = 50, 22.1%) and no (n = 9, 4.0%).

#### 3.1.2 Citizen science background

**Figure 4:**
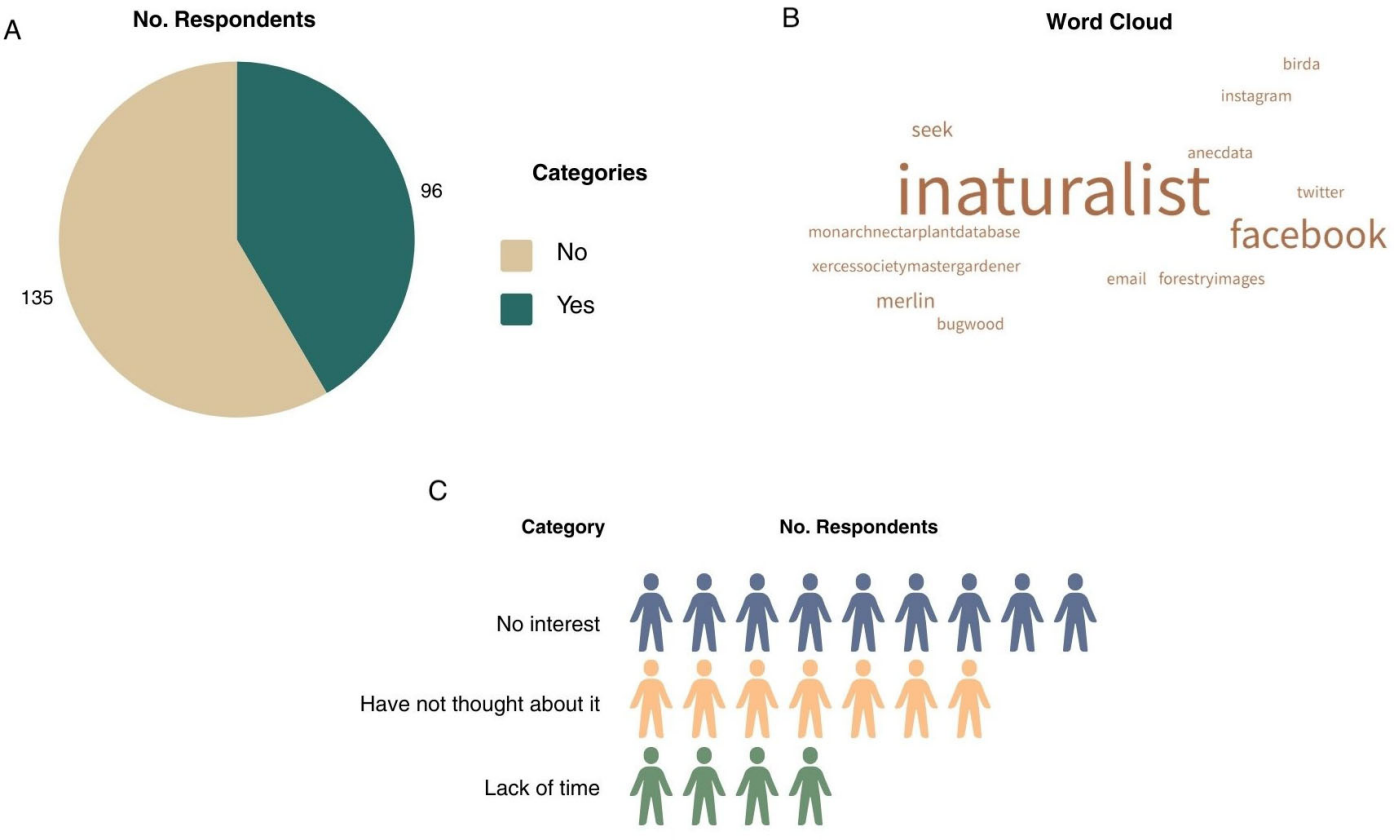
**(A)** Question 5 (Q5) data on if survey respondents have ever shared nature photos online. **(B)** Question 6 (Q6) data on what platforms respondents submit photos to. **(C)** Question 7 (Q7) categorized data on why respondents have not submitted nature photos online.

In Question 5 (Q5), participants were asked about prior engagement in sharing nature photographs online. 231 responded with the majority reporting ‘no’ (n = 135, 58.0%), whereas 96 participants (42%) indicated ‘yes’ (**Figure 4**). In the open-ended follow-up question (Q6) asking participants who responded “yes” to specify the platforms where they shared photos, 20 participants responded with iNaturalist being the most frequently mentioned platform. In a subsequent open-ended question (Q7) directed to participants who responded “no,” 20 participants responded and three categories were identified: (1) no interest, (2) have not thought about it, and (3) lack of time. The most common explanations for not sharing nature photographs were a lack of prior consideration or interest. Included in ‘no interest’ were participants stating that they did not feel they were qualified to participate in citizen science projects. Also in category 2, some participants stated that they did not know these programs or platforms existed. Others indicated limited time to contribute or to learn how to engage with such platforms.

#### 3.1.3 Willingness to participate

**Figure 5:**
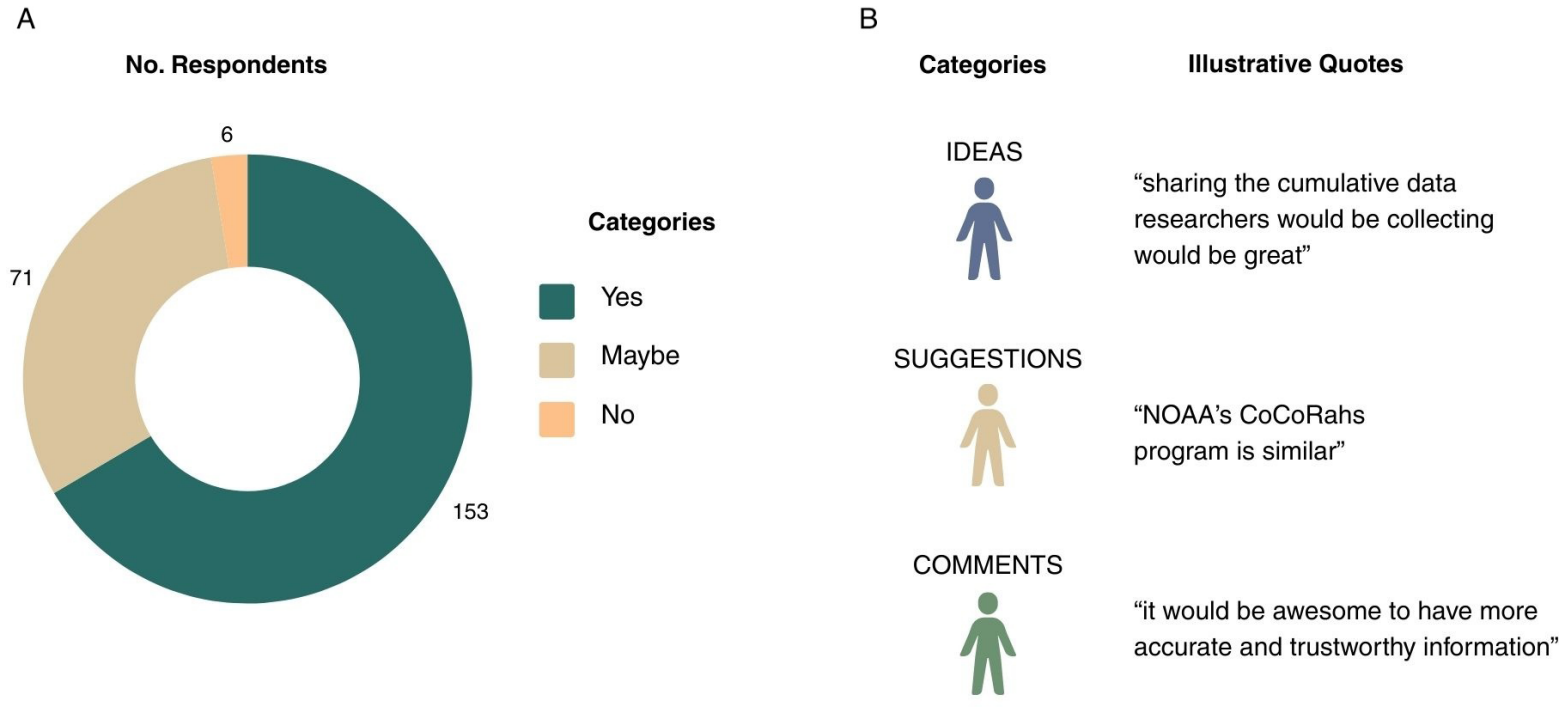
**(A)** Question 8 (Q8) data on if survey respondents would like to contribute to a citizen science project on plant disease monitoring. **(B)** Question 9 (Q9) categorized data on respondents’ ideas, suggestions, and comments to a plant disease citizen science project.

In question eight (Q8), participants were asked whether they would be willing to share plant disease sightings with researchers if an appropriate platform were available. Of the 230 respondents, a total of 153 respondents (65.7%) selecting ‘yes’, 71 (30.5%) selecting ‘maybe’, and 6 (2.6%) selecting ‘no’ (**Figure 5**). In the final open-ended question (Q9), which invited participants to share comments or suggestions regarding citizen science in plant disease research, 23 respondents answered with the predominant theme being a desire for greater access to plant disease information prior to contributing photos or data. Participants emphasized the need for region-specific disease resources, accurate identification tools, and an accessible reporting platform. Overall, most respondents expressed an interest in expanding their plant disease knowledge to enhance the quality of their potential contributions.

## 4 Disscusion

Referring to the three general goals listed in the experiment design portion of this project: (1) to assess participant’s plant disease knowledge, willingness to participate, and citizen science background, (2) to identify what obstacles exist to integrating citizen science in the field of plant pathology, and (3) to identify best methods of utilizing and supporting citizen science in plant disease surveillance; (1) 81% of participants reported to have greater than intermediate knowledge on plant diseases and 74% selected they want to learn more on plant diseases, while 22% selected “maybe”. Despite 58% of participants having no prior citizen science background, only 3% said they would not contribute to a citizen science project. (2) As indicated in previous studies looking at obstacles to integration with citizen science in plant disease detection, accurate diagnosis was previously deemed the most prevalent obstacle (Barbedo, 2016). The results of the present study showed that some citizen scientists were aware of this primary concern and would like some level of professional confirmation conducted to ensure data is correct. (3) Participants indicated that building upon an already-existing platform would be the best approach to meet all the criteria and additional challenges to creating a citizen science plant disease project. If someone wanted to create a novel project or platform, however, the incorporation of educational materials, a ‘train the trainer’ style format, and a form of professional validation were indicated as elements that would most contribute to a successful outcome.

The results show that there is high interest from citizen scientists to be involved with and contribute to plant disease data. Participants of the survey acknowledge existing difficulties with contributing to research data, and that more information would be needed on the citizen scientists’ part to be able to confidently and accurately contribute to research data in a meaningful way. Participants also emphasized the importance of a simple to use platform as well as a desire to have more accessible information on plant diseases. Our findings extend and contribute to previous work on citizen science, specifically passive surveillance, in plant health by Brown et al. (2020). This study examined various forms of passive surveillance used in detecting tree pathogens, addressing their underlying principles, current challenges, and potential applications in tree health monitoring. The work further provides recommendations for advancing the development and effective utilization of passive surveillance methods and data. This current study supports findings by Brown et al. (2020) by offering needs as explained by citizen scientists, that directly align with literature findings from previous study.

The results of this current study, as revealed in the final open-ended question on additional suggestions, have mixed reactions about integrating an app such as iNaturalist for citizen science activities related to plant disease monitoring. Although mobile applications provide an accessible platform for data sharing, accurate plant disease diagnosis often requires substantial supplementary information beyond photographs, which can be influenced by user bias. A previous study reviewing the effectiveness of an Artificial Intelligence and Deep Learning application specifically for citizen science use in detecting plant diseases (PestNU, n.d), concluded the platforms effectiveness in identifying *Tuta absoluta* in greenhouse grown tomatoes in Murcia, Spain (Christakakis et al., 2024). While this study was conducted with a sample size of 50 in a controlled setting, the potential of an app to gather and organize data on multiple different causal agents across a large geographic region still remains in question. An additional deep learning study by Boyar and Yıldız (2022) employed the YOLOv5 (Ultralytics, n.d) model to detect powdery mildew in hazelnut plants. Although the study was limited by a relatively small dataset, it ultimately came to the same conclusion of the previously mentioned study, indicating that while image-based approaches show promise for plant disease detection, they do not yet constitute a fully reliable or robust diagnostic method. Another factor to be considered when developing a new app is user adoption, which may be relatively small when focused solely on a niche field such as plant disease. However, user adoption may be increased in these independent apps when considering one of the pressing needs revealed in the current study, increased assessable disease information. By integrating an educational aspect into independent diagnostic apps, citizen scientists have the ability to increase plant disease awareness and add personal diagnostic input.

Considering both previous research on citizen science and the responses from participants in the present study, the development of effective programs can draw valuable insights from existing successful models. One such project highlighted by study participants is the CoCoRaHs program sponsored by NOAA. This program uses citizen science observations to map and measure rain, hail, and snow in local communities (CoCoRaHs, n.d). With over 27,000 volunteers, the program’s website is a combination of educational resources, videos on how to participate, state newsletters, and results. Another successful program highlighted by participants is FrogWatch, sponsored by Connecticut Beardsley Zoo (Beardsly Zoo, n.d). FrogWatch, similar to CoCoRahs, has a variety of educational materials and also a training program. A recent systematic literature review on computer-based plant disease detection methods further emphasizes the importance of incorporating training programs for stakeholders to ensure the effective and accurate use of these emerging systems (Yilmaz et al., 2025). A ‘train the trainer’ style inclusion which has proven successful in established citizen science programs is crucial when looking to utilize citizen scientists in a field where information is needed beyond photographs. Previously, the First Detectors Program, funded by the USDA, was this exact program for plant disease (NPDN, 2025). The program has since ceased operations, however utilizing the foundations from that program into a smaller scale or project specific method would prove an invaluable resource for the progression of citizen science style projects in the field of plant health.

Although this project offers meaningful perspectives on how citizen scientists view plant disease data, it is important to recognize certain limitations that may influence the interpretation of findings. As the survey was primarily disseminated through extension communication networks such as university staff pages, national organizations email lists, and LinkedIn pages, most respondents had roles which were heavily integrated in plant science, causing potential bias throughout survey results. No geographical data was collected from participants, which limits the ability to assess regional trends or tailor future engagement strategies. Such data would have been valuable for identifying and interacting with geographically focused groups expressing greater interest in contributing to plant disease research. Finally, in question 3 (Q3), 41 respondents selected ‘other’ as a means of learning about plant disease. A follow-up clarification question on what the described unknown resources for learning about plant diseases are could have provided insight on possible educational routes to be utilized. In future studies, if this project is to be re-executed, it is recommended to address these noted limitations.

For recommendation on future studies to build upon this work, a follow-up project, tailored to citizen scientists not currently in an agricultural role, would play a vital role in understanding the true scope of potential within a plant disease citizen science project. Such as disseminating a similar or the same survey to groups that are not explicitly related to plants or agriculture. For a project of this type, it would be valuable to identify whether there are additional interest groups, beyond those already studied, that demonstrate greater motivation or willingness to participate in a plant disease–focused citizen science initiative. Additionally, a complimentary project identifying or collecting geographical data on participants interested in contributing to data would be critical market research needed to accurately test citizen science projects with minimal bias. Similar in format to the project suggested above, this survey would be disseminated to a larger audience and include the collection of state and county geographic data. The project output could potentially be a heat map displaying areas of the country which have the most participant interest and therefore potential as a starting point for a citizen science initiative.

For additional recommendations on implementing citizen science in plant disease research, we believe that further developing an existing platform such as iNaturalist would be the easiest method to gain the quickest and most widespread user adoption. If looking to create a new platform or project, the most successful projects should contain a large educational aspect and some form of non-rigorous training. For user tasks within plant disease data, it would be best to incorporate geographic location and additional questioning (plant species and cultivar, surrounding area conditions, possible plant history) with photo submissions. Until AI is more thoroughly validated for plant diagnostics, a trained diagnostician should validate findings. Additionally, citizen science projects focused on plant disease data should be centered around tracking and the movement of diseases, not providing diagnostic management. Finally, interactions with citizen scientists through newsletters, regional data, or new research, were noted as additional value points for potential users.

## 5 Conclusion

As plant diseases continue to threaten global agriculture and horticulture, it is crucial to implement new methods and technics to more efficiently track the movement of diseases. This paper surveyed 233 citizen scientists with the goal of identifying obstacles and best implementation methods of citizen science in plant disease data. The results showed that an overwhelming majority of participants are interested and willing to contribute to citizen science projects on plant health. Further results showed similarities in the perceived requirements for a plant disease citizen science project from past studies, and from what citizen scientists directly state they would need to see to be willing to contribute to a project. In addition to previous perceived needs, results also showed specialized value points such as a ‘train the trainer’ program incorporated into projects, general educational materials, and professional validation. The limitations of this project included unintended role specific participant background, lack of participant geological data, and lack of specification for one of the questions. In conclusion, this research provides the first user targeted evidence that citizen science in plant disease data is a viable route forward, it also lays out recommended actions and implementations for anyone looking to create a plant disease specific successful citizen science program.

## Data Availability

The original contributions presented in the study are included in the article/supplementary material. Further inquiries can be directed to the corresponding author.
